# Electrooxidation of Hypercoordinated Derivatives of Silicon and Reactivity of Their Electrogenerated Cation Radicals: 1-Substituted Silatranes

**DOI:** 10.3390/molecules28145561

**Published:** 2023-07-20

**Authors:** Vitalijs Romanovs, Elena F. Belogolova, Evgeniya P. Doronina, Valery F. Sidorkin, Viatcheslav V. Jouikov

**Affiliations:** 1 Latvian Institute of Organic Synthesis, Aizkraukles 21, LV-1006 Riga, Latvia; 2 A. E. Favorsky Irkutsk Institute of Chemistry, Siberian Branch of the Russian Academy of Sciences, 1 Favorsky Str., 664033 Irkutsk, Russia; lena@irioch.irk.ru (E.F.B.); evgdor.irioch@gmail.com (E.P.D.); 3 UMR 6226—Institute of Chemical Sciences of Rennes, University of Rennes, 35042 Rennes, France

**Keywords:** silatranes, cation radicals, hypercoordination, electrochemistry, EPR spectroscopy

## Abstract

Electrochemical oxidation of 1-R-substituted silatranes **1** (R = Me, vinyl, (CH_2_)_2_CN, CH_2_Ph, CH_2_(C_10_H_7_), Ph, C_6_H_4_Me, *p*-Cl-C_6_H_4_, Cl)—classical representatives of pentacoordinated silicon compounds—and the formation of their short living cation radicals upon reversible or quasi-reversible one-electron withdrawal were studied by means of cyclic and square-wave voltammetry, faradaic impedance spectroscopy and real-time temperature-dependent EPR spectroelectrochemistry supported by DFT B3PW91/6-311++G(d,p) (C-PCM, acetonitrile) calculations. The main reaction responsible for the decay of **1**^+•^ is shown to be their deprotonation, and ways of increasing the stability of these species are proposed.

## 1. Introduction

Since the discovery of triptych-siloxazolidines [1], later coined silatranes, they have attracted a great deal of interest from researchers, which has been documented in more than 1300 publications. However, during the first peak of silatrane research between 1970 and 1980, when they were studied using virtually all of the physico-chemical methods then available [2,3], organic electrochemistry only began to expand into organometallic chemistry [4], with only two closely related accounts appeared in the early 1990s [5,6], covering the electrochemical oxidation of silatranes. The peak potentials *E*_p_ of several 1-R-substituted compounds were then reported, and a putative mechanism, involving H_2_O and a progressive cleavage of the side chains of the atrane ring, was suggested. The idea of specific intramolecular 3c-4e bonding in these compounds had already been advanced by that time [7], so the authors considered the process through that prism, concluding that “silatranes undergo electro-oxidation without preceding cleavage of donor-acceptor bonding N→Si” [6].

It was not until 2016 that there was a resurgence in the interest in molecular electrochemistry of silatranes [8], occurring due to the rising interest in long multi-electron multi-center hyperbonds [9] beyond the century-old Lewis 2c-2e bonding model [10], and to the specific properties conveyed to silatranes by the 3c-4e internal N→Si bond, in particular, which rendered them promising for the development spintronic and electromechanical molecular systems for the development of a new generation of functional interfaces and molecular wires [8,11,12]. 

The formation of transient cation radicals (CRs) from the electrooxidation of silatranes [8,11,12,13,14] and of homologous germa- [15,16,17] and stannatranes [12,18], as well as of the related silocanes [19,20] has been reported within last 5–7 years. Yet the question as to whether the formation of CRs is a universal feature of silatranes or represents a particular case, as well as the problem of their reactivity and the factors determining their stability, remain to be addressed. 

**Scheme 1:**
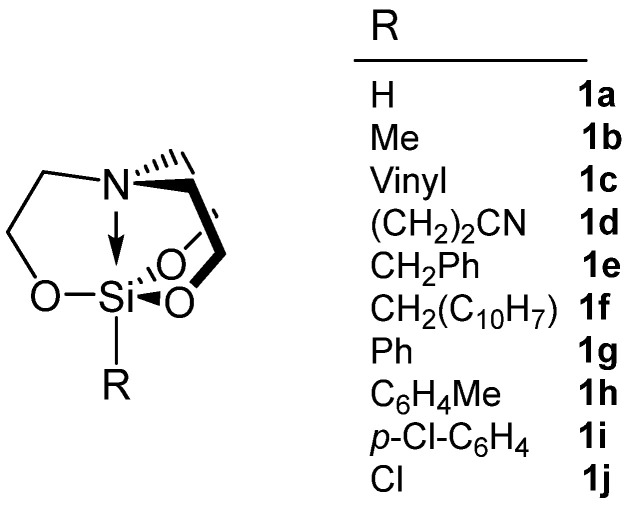
The silatranes considered in this study.

The present communication aims to further consider these problems by means of a palette of electrochemical techniques, including cyclic voltammetry (CV), square-wave voltammetry (SWCV), electrochemical impedance spectroscopy (EIS)—the classical method for which allows the Nernstian nature of electron transfer (ET) to be established and the mechanism and its quantitative characteristics to be assessed from a formal kinetics point of view—as well as real-time EPR spectroelectrochemistry, enabling direct observation of the CRs in order to provide insights into the spin distribution and electronic structure of such species, and supported by computational study of the reaction series of the following silatranes (Scheme 1). 

## 2. Results and Discussion

### 2.1. Electrochemistry

#### Formation of CRs 

Due to the specific orbital structure of **1** involving two electronically interacting units—the atrane cage and the substituent R—the withdrawal of an electron from **1** may affect three different centers [14]. Two possible sites of ionization, which have also been shown to be heavier congeners of **1** (with M = Ge [17] and Sn [18]), are the atrane moiety (the 3c-4e system with the N lone pair as a main donor component) or the substituent at Si, depending on the proper ionization potential (IP) of the corresponding unit. Recently, a third site of oxidation via electron removal from the σ_(Si-Y)_ bond was theoretically predicted [14] (cf. [13]). In some cases [14], an interconversion of the sites of ET between the atrane cage and the 1-R substituent can occur when varying the N**^…^**Si distance. We shall therefore focus on the oxidation and formation of CRs of “proper” silatranes **1** only, i.e., of those whose HOMO is located on the atrane unit. 

The formation of CRs of parent trialkylamines, although not always experimentally observed [21,22], had already been suggested in earlier works [23,24,25]; deprotonation, as the main reaction of such CRs [21,22,26,27,28,29,30], was subsequently well established [31,32,33,34,35]. For several silatranes, oxidation has also been reported to be reversible [11,13,14], although their CRs appeared to be very short-lived.

**Figure 1:**
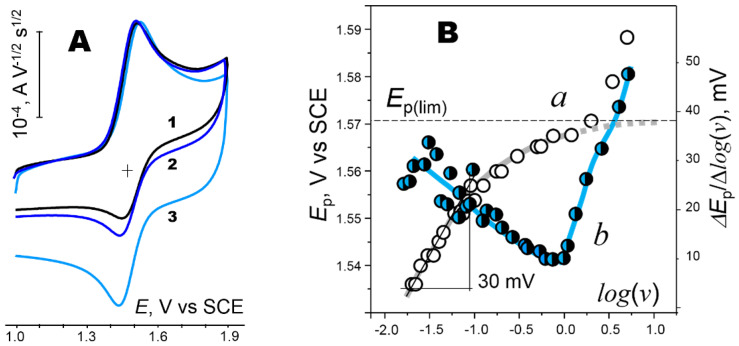
(**A**) Scan–rate–normalized voltammograms of **1h** at a Pt electrode in CH_3_CN/0.1 M Bu_4_NPF_6_. Scan rates *v*: (1) 0.1, (2) 0.2 and (3) 1 V s^−1^. The cross denotes *E*_0_. (**B**) Variation of *E*_p_ of **1h** with changes in scan rate. (a) Experimental (circles) and expected *E*_p(lim)_ (*E*_0_ + 28.5 mV [27]). (b) Derivative of *E*_p_ − *log*(*v*) plot (Δ*E*_p_/Δ*log*(*v*) vs. *log*(*v*)).

**Figure 2:**
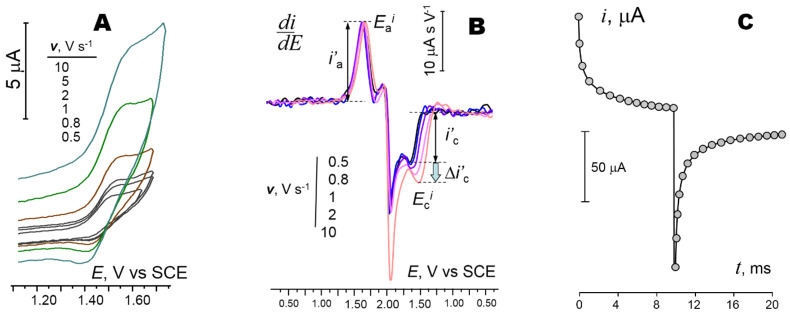
Oxidation of **1d** (2 × 10^−3^ mol L^−1^) at a Pt electrode in CH_3_CN/0.1 M Bu_4_NPF_6_. CV (**A**) and normalized derivative CV (**B**) at different scan rates. *E*^i^_a_ and *E*^i^_c_ are inflection potentials for anodic and cathodic scans; note that the X-axes represent the *E*(*t*) inverted at the vertex potential (*E* = 2 V) and correspond to the time course during the scan. (**C**) Two-step potentiostatic chronoamperometry (CA): experimental (solid line) and simulated (circles) results using the following parameters: *k*_s_ = 4.6 × 10^−2^ cm s^−1^, *k* = 0.66 s^−1^, α = 1.857(RT/FΔ*E*_p−p/2_); *t*_1_, *t*_2_ = 10 ms.

**Figure 3:**
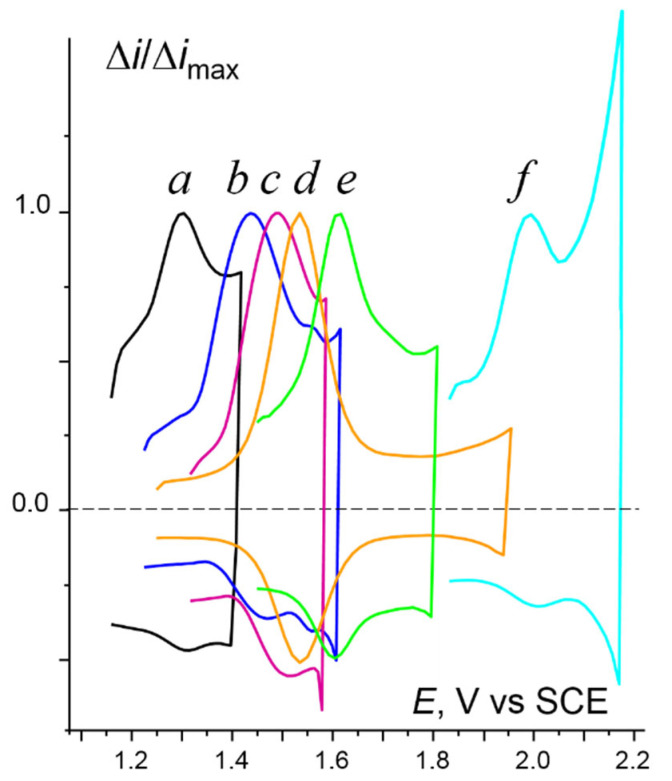
Current-normalized square-wave CV of (a) **1e**, (b) **1b**, (c) **1c**, (d) **1h**, (e) **1i** and (f) **1j**. Pulse 25–50 mV per (2–3) × 10^−2^ s, step height 15–20 mV, *v* = 0.2–1 V s^−1^ (*v* = 5 V s^−1^ in (e)). CH_3_CN/0.1 M Bu_4_NPF_6_, Pt electrode.

For most of the silatranes studied, the formation of **1^+^**^•^ could be unambiguously shown by cyclic voltammetry: the one-electron stoichiometry of the first oxidation step (n = 1, from both *i*_p_*v*^−1/2^/*it*^1/2^ and *i*_p_(**1**)/*i*_p_(Fc) criteria [27]) and the reduction peak of the CRs (**1c-d**, **1h**, **1i**, Table 1), appearing at different scan rates on the reverse scan, were evidenced (Figure 1 and Figure 2). This is in agreement with the general scheme of EC oxidation (electrochemically reversible ET followed by a fast chemical reaction consuming the primary CRs). For other silatranes, the CRs were less stable, but their formation could still be demonstrated by two-step potentiostatic chronoamperometry (CA) (Figure 2C) or by square-wave cyclic voltammetry (SWCV) (Figure 3). Indeed, while in CV [36], the CRs produced during the forward scan had to wait in the near-to-the-electrode area for the reverse scan to reach the *E*_p_ of the backward ET process (while diffusing to the bulk solution and being consumed in the follow-up reactions), the potential in SWCV [36] is incremented in small steps around ET equilibrium, making it possible to overcome this limitation of CV. 

**Figure 4:**
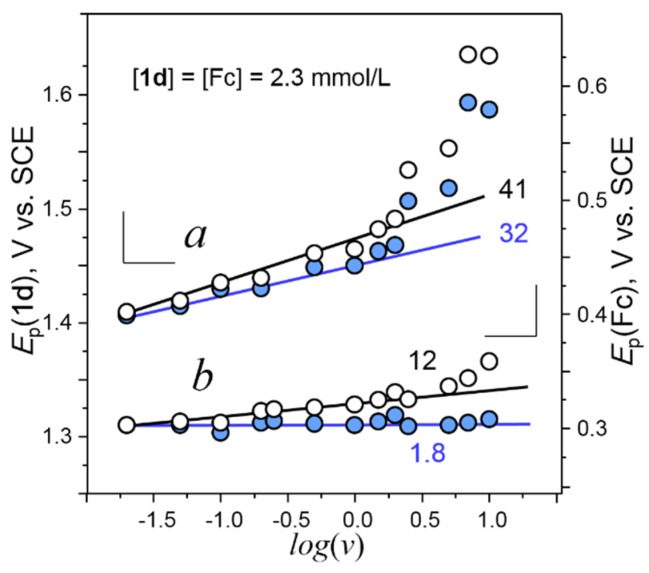
*E*_p_ − *log*(*v*) plots for the oxidation of **1d** (a) and Fc (b) at a Pt electrode in CH_3_CN/0.1 M Bu_4_NPF_6_. Hollow circles: experimental points (*E*_p_/*log*(*v*), mV/dec, black numbers); blue filled circles (blue numbers): IR-corrected to *E*_p_(Fc)/*log*(*v*) ≅ 0 mV and Δ*E*_p_^a-c^(Fc) = 60 mV.

The above *v* ≅ 1 V s^−1^, peak half widths *E*_p_ − *E*_p/2_ (Table 1) and, when applicable, *E*_p_^ox^ − *E*_p_^red^ separations exceed those of a purely Nernstian process. This might be caused by the adsorptive interactions of silatranes occurring due to their high polarity [2,37], or by undercompensated solution ohmic drops IR [38]; however, the rapid increase in these entities with *v* can also be indicative of the limitations of ET kinetics by a skeletal reorganization accompanying the process. A ferrocenium/ferrocene rapid redox system with *k*_s_(Fc) ≥ 6 cm s^−1^ [39] and Δ*E*_0_ ≠ f(*v*) [40] was used under similar conditions as a reference for the correction of IR drops (Figure 4). 

The half width of the oxidation peaks (Δ*E* = *E*_p_ − *E*_p/2_) of **1**, providing (through α and *k*_s_ [27]) the information on ET kinetics, was also systematically higher (≈70–100 mV, except for **1e**, Table 1) than the 58 mV of a purely Nernstian process [27]. This difference, remaining even after IR correction, was considered to arise from important structural changes induced by ET (the onset of the ET control at *v* = 2–3 V s^−1^ can be seen more clearly now, Figure 4), such that their oxidation can be qualified as being quasi-reversible.

**Figure 5:**
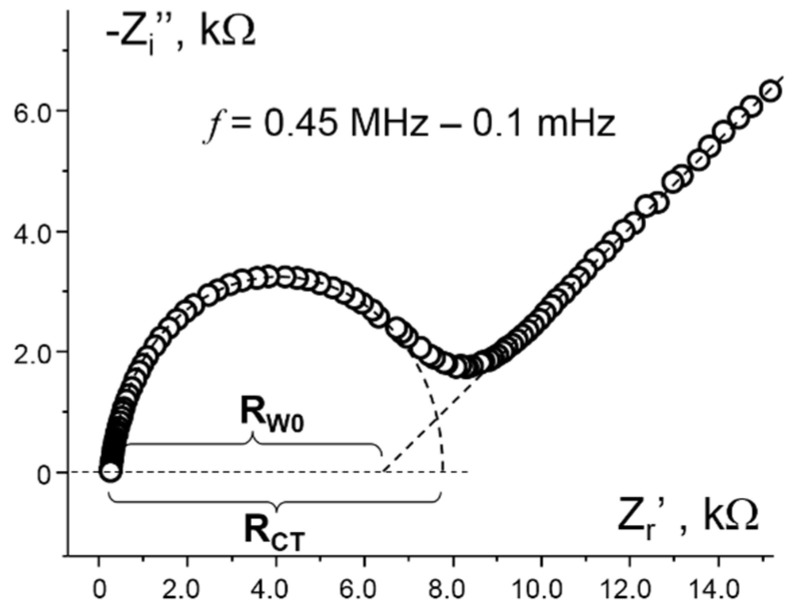
Nyquist plot of the oxidation of **1c** (1 mmol L^−1^) in CH_3_CN/0.1 M Bu_4_NPF_6_. E = 1.5 V, ΔE = 10 mV. Charge transfer resistance R_CT_ = 7.2 kΩ, diffusion-free resistance R_W0_ = 5.9 kΩ and double layer capacitance C_DL_ = 5.14 × 10^−9^ F, *k*_s_ = 0.078 cm s^−1^.

In cases where the CV signal was too poorly shaped to be able to draw any reliable conclusions, electrochemical impedance spectroscopy (EIS) [41] was used to assess the rates of ET (Figure 5, Table 1) at the apparent *E*_0_ and a small modulation of ±10 mV. The obtained *k*_s_ values vary in the range (0.3–27) × 10^−2^ cm s^−1^ (Table 1), which is in good agreement with the results of CV. Indeed, with the reversibility of the first step of oxidation attained in CV at *v* = 2–5 V s^−1^ and α from Δ*E*_p−p/2_ = 1.857(RT/αF), the ET rate constants, estimated on the basis of *k*_s_ ≅ (αF*v*D/RT)^1/2^ [38], amount to (0.6–4) × 10^−2^ cm s^−1^. These values are also comparable with those previously reported for germatranes [17].

Of the variations in the characteristic parameter Δ*E*_p_/Δ*log*(*v*) with changing scan rate (Figure 1B) show a tendency toward Δ*E*_p_/Δ*log*(*v*) → 0 (to a purely Nernstian ET, by extrapolation expected at *v* ≅ 10 V s^−1^), being drastically limited by the rate of ET kinetics at its onset at *v* ≅ 1 V s^−1^. 

The *k*_s_ of **1** seems to be at the lower end for a reversible ET, but this can be rationalized by keeping in mind a specific space-distributed nature of the 3c-4e HOMO of silatranes, the extreme sensitivity of the N→Si distance to the media effects [2,42], and to the fine orbital interactions up until HOMO inversion [14], yet limited by the time scale of relaxation of the atomic carcass of the atrane unit. The polarization of this complex orbital system along the ET reaction coordinates causes breakage of the 3c-4e intramolecular bond (thus conveying a dissociative character to the process) and forces the atrane core in the CR to adopt a geometry substantially different from that of **1**. This entails a substantial contribution of this reorganization (λ = λ_0_ + λ_i_ + D_N→Si_) to the process free energy ΔG^≠^, which directly affects *k*_s_. 

Thus, two regimes can be evidenced for the oxidation of **1**: (i) an electrochemically reversible ET with a moderately fast follow-up reaction (EC-scheme) at a slow scan rate *v*; and (ii) an ET whose rate is limited by the carcass reorganization of the atrane cage, at *v* ≥ 2–5 V s^−1^.

For the same ET reaction site (i.e., the atrane unit), the rate of ET in the series **1** is substantially modulated by the substituent R at silicon (Table 1). Therefore, the length of the N→Si contact in the atrane cage (*l*_Si-N_) is directly related to the degree of the involvement of the nitrogen lone pair in the 3c-4e system, and hence to the ease of electron withdrawal (IP and HOMO level). Therefore, the variation of *l*_Si-N_ caused by the oxidation (destroying the N→Si coordination in **1**^+•^ [11]), i.e., Δ*l* = *l*_Si-N_(**1**^+•^) − *l*_Si-N_(**1**) (Table 2) is expected to correlate with the reorganization energy of the atrane carcass (λ_i_), and further with *k*_s_. Then, shifting the process to the zone of ET control (*v* > 1–2 V s^−1^, the rising parts of the Δ*E*_p_/Δ*lg*(*v*) plots (see, e.g., Figure 1B) makes it possible to derive ΔG_0_^≠^ from the slope α/*E*_p_ [38] and to consider the variation in the ET rate *k*_s_ with the structural changes (*l*_Si-N_) during the oxidation of silatranes. Indeed, with the exponential character of the *k*_s_ = *f*(λ) dependence (through *k*_s_ = Z_het_exp(−ΔG^≠^/RT) and ΔG^≠^ = (λ/4)(1 + (*E*_p_ − *E*_0_)/λ)^2^ [38], where Z_het_ is the electrochemical heterogeneous collision factor), this can be well seen (Equation (1), Table 1 and Table 2) through the good correlation of *log*(*k*_s_) with Δ*l*_Si-N_ (n = 7, R = 0.99, *p* < 0.0001):

*log*(*k*_s_) = −19.443 × Δ*l*_Si-N_ + 15.941 (1)

Therefore, it can be stated that the CRs are subject to a skeletal rearrangement towards the *exo*-configuration of N atom geometry related to the destruction of the N→Si bond upon oxidation, because after the removal of one electron from the 3c-4e bond, the remaining 3c-3e system is devoid of any bonding character.

It should be noted that, by means of CV, the interconversion **1**_exo_^+•^/**1**_endo_^+•^ and the appearance of a new redox system related to the *exo*-form **1**_exo_^+•^ (with broken N→Si bond) could only be discerned on the basis of formal voltammetric criteria. Interestingly, for oligosilylated silatranes [13], silocanes [20] and tribenzsilatranes [43], CV revealed the appearance of new Nernstian redox couples (with *E*_0_ < *E*_0_(**1**)) formed upon the withdrawal of one electron from **1** that were ascribed by the authors to an *exo*-form of **1**^+•^ (in other terms, a *long*-bond isomer of **1**^+•^ [14]), related by reduction by one electron to a neutral species formed without a detectable structural reorganization, formally **1**_exo_ (i.e., supposedly a *long*-bond isomer of **1**). In line with the above is the complexation of ferrocene with a donor form of **1**, issued from the reduction of **1**^+•^, observed by CV for silatranes [14] and silocanes [19]. However, this contrasts with the results of quantum chemical calculations [14], whereby a sole *endo*-form of the neutral **1** was localized on its potential energy surface; hence, all ET reactions of **1** are supposed to involve **1**_endo_ only. Note that in such a case, an **1**^+•^_exo_/**1**_endo_ (i.e., *long*–*short*) couple would have the markedly different feature of a quasi-reversible ET, where the secondary oxidation peak would appear at *E*_p_(**1**). At this point, it is hard to entirely rationalize this situation (see Section 2.1).

### 2.2. EPR Spectroscopy

Since electrochemistry is an indirect method solely attesting the occurrence of ET processes regardless of the reaction site, the electrochemical kinetic analysis only permits establishing a formal reaction mechanism, while saying nothing on the nature of the species involved. Coupling this with EPR spectroscopy dramatically enhances its capacity, allowing the distribution of electron density to be studied directly in odd-electron species produced by ET, thus establishing the reaction site. Thus, the EPR signature of the electrogenerated CRs unambiguously enables sorting out the “proper” from the “non-proper” silatranes in the redox context.

Real-time EPR-spectroelectrochemistry, when **1**^+•^ are in situ electrogenerated in the cavity of the EPR spectrometer, reveals two types of spectra of the species resulting from the oxidative ET.

#### 2.2.1. “Proper” Silatranes **1**

**Figure 6:**
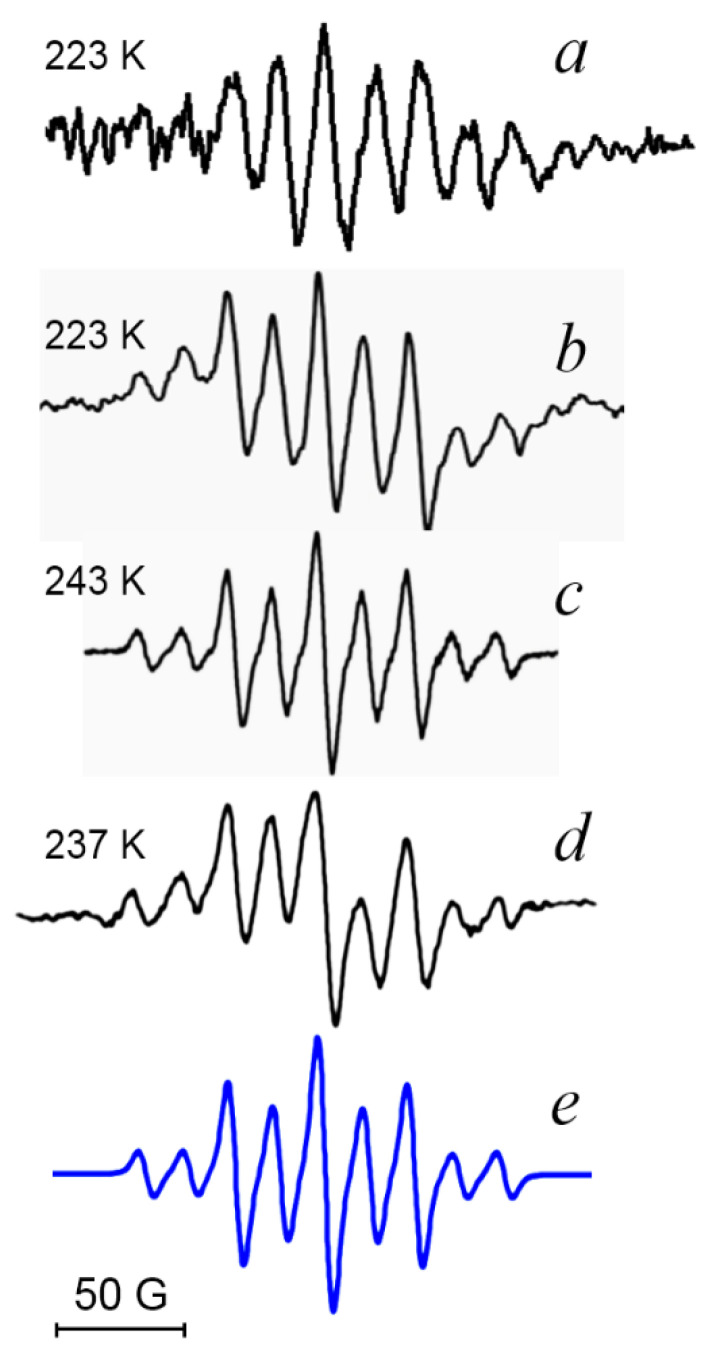
EPR spectra of (**a**) **1b**^+•^, (**b**) **1c**^+•^, (**c**) **1g**^+•^ and (**d**) **1h**^+•^ electrogenerated at a Pt micro spiral electrode in CH_3_CN/0.02 M Bu_4_NPF_6_. Frequency, 9.46 GHz; modulation, 100 Hz. (**e**) Generic spectrum of an atrane-localized radical.

**Figure 7:**
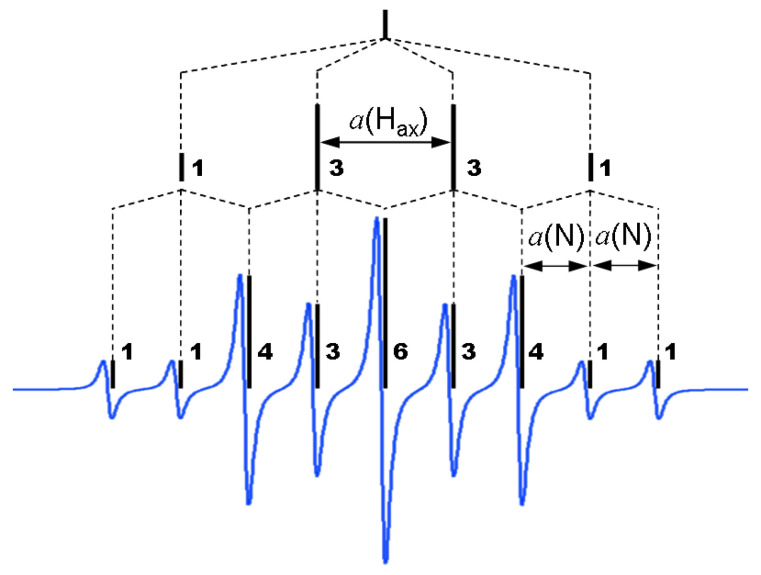
Schematic representation of the signal splitting at the N and H nuclei in the EPR spectra of “proper” silatranes. Smaller *hfc* constants from equatorial α-H_eq_ and β-H protons are not shown. The numbers correspond to the resonance line integration intensities.

When dealing with the oxidation of proper silatranes (i.e., when IP_atrane_ < IP_substiruent_), the unpaired electron in the CR is mostly localized on the practically planar N atom of the atrane moiety, and their large EPR spectra, which have an end-to-end width of ca. 200 G (recorded at T = 223–263 K; the temperature was adjusted for each **1** in order to stabilize its electrogenerated CRs—lower T for less stable CRs), consequently show the nine-line generic features characteristic of atrane cage radicals (Figure 6, cf [11,17]) with quite similar N *hfcc* (*a*_N_ = 18.54, 18.60, 18.41 and 18.71 G for **1b**^+•^, **1c**^+•^, **1g**^+•^ and **1h**^+•^, respectively) and a large (*a*_H_ = 36–37 G) coupling with the three axial protons of α-CH_2_ groups. According to Pascal’s triangle, splitting from three α-H protons (*I* = 1/2) gives four resonance lines with an intensity ratio of 1:3:3:1, which, interacting with the N nuclei (*I* = 3/2), ultimately leads to a generic 1:1:4:3:6:3:4:1:1 pattern (Figure 7). The angle Θ formed by the axially oriented α-C-H bonds relative to the spin-carrying (N)p_z_ orbital in **1**^+•^ can be estimated through *a*_H_ = B cos^2^Θ using *a*_H_ = 28.56 G, <cosΘ> = ½ and B = 57.1 G for the rotation-averaged protons of (CH_3_)_3_N^+•^ cation radical in solution [44] and *a*_H_ for **1**^+•^ (Figure 6). This angle in **1**^+•^ (even in solution, the space orientation of α-H protons in the atrane cage is fixed due to the rigidity of the latter) obtained from EPR (Θ = 19°) is then in very good agreement with that obtained from DFT calculations (Θ = 21.56°). The generic spectrum of these silatranes is practically identical to that of manxine (1-azabicyclo [3.3.3]undecane) recorded at 240 K (*a*_N_ = 19.3 G, *a*_H-α_ = 38.5 G [45]), which has a rigid carcass shaped similarly to that of silatranes. These common features attest to a similar flattening of the atrane N atom in manxine CR and in **1**^+•^ and to the existence of the two sets of non-equivalent α-H atoms, which are different in their orientation with respect to the spin-carrying N(p_z_) orbital (with *hfcc*(α-H_axial_)/*hfcc*(α-H_equatorial_) ≅ 42–45), and therefore to the similar spin distribution in these species. Note that the related Et_3_N^+•^ does not have a rigid carcass, and exhibits an entirely different EPR spectral signature (*a*_N_ = 20.8 G, 6 × *a*_β-H_ = 19 G [46]); the pyramidal structure of Et_3_N^+•^ was confirmed experimentally [47].

#### 2.2.2. “Non-Proper” Silatranes: Naphthylsilatran **1f**

**Figure 8:**
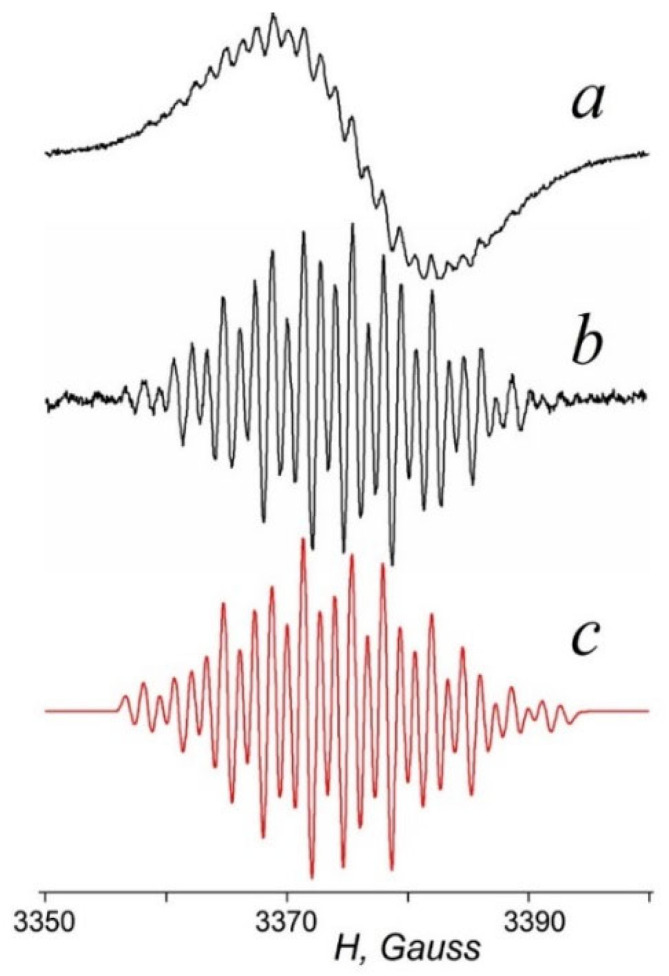
EPR spectrum of the CR of naphthylsilatran **1f**^+•^ electrogenerated at *E* = 1.27 V in CH_3_CN/0.05 M Bu_4_NPF_6_ at a Pt micro spiral electrode. T = 235 K. (**a**) Raw experimental spectrum, (**b**) FFT filtered, and (**c**) simulated. Spectrum width, 37.2 G; *g* = 2.0047; *a*_N_ = 6.151 G, 2 × *a*_H1_ = 6.232 G, *a*_H2_ = 2.365 G, *a*_H3_ = 0.357 G, *a*_H4_ = 3.661 G, *a*_H5_ = 1.361 G, *a*_H6_ = 0.203 G, *a*_H7_ = 0.180 G, *a*_H8_ = 1.348 G.

The EPR spectrum of the electrogenerated CR of naphthylsilatrane **1f**^+•^ (Figure 8) has a remarkably smaller, ca. 40 G, end-to-end width, with a *g*-factor slightly higher than that of most of aryl radicals (typically 2.002–0.0025 [48]), and the *hfc* constant of the methylene protons is 2 × *a*_H1_ = 6.232 G (compared to 3.87 G in the anion radical of methylnaphthalene [48]); the other *hfc* constants of **1f**^+•^ are close to those for naphthylmethyl, phenylnaphthalene and 1,2-di(1-naphthyl)-ethene ion radicals [48].

A small *hfcc* of an N atom might be interpreted as the oxidation affecting the naphthyl unit (IP_naphthyl_ < IP_atrane_), which consequently obtains a positive charge, enhancing its electron-withdrawing character. It is translated by the reinforcement of the N→Si 3c-4e dative interaction, as is observed upon increasing electronegativity of the substituents at Si in neutral silatranes [2] or when their neutral substituent becomes positively charged [49]. This is in agreement with the concept of conservation of the total bond order around Si [50], and with the oxidation features observed for diorgano *bis*-germatranes [51]. Since the atrane unit is not affected by ET, this type of CR is then of a “non-proper” silatrane type.

### 2.3. Follow-Up Reactions of Silatrane CRs

#### 2.3.1. Monomolecular Decay of **1**^+•^

As attested by the invariance of the Randles-Ševčík parameter *i*_p_/C*v*^1/2^ (with *log*(*i*_p_)/*log*(*v*^1/2^) = 0.46–0.48) within the range of concentrations C = 0.1… (7–8) mmol L^−1^ and *v* > 0.05 V s^−1^, the first step of the oxidation of silatranes **1** is diffusion-controlled under these conditions. For **1c**, **1g** and **1d** within 270 < T < 310 K, the Arrhenius slope *log*(*i*_p_)/(1/T) for the peak current *i*_p_ does not exceed the activation energy of viscous flow in CH_3_CN (6.12 kJ mol L^−1^ [52]), which also confirms the diffusional nature of *i*_p_ and the absence of any kinetic (of either the CE or ECE type [27,36]) components in the process. Invariant electron stoichiometry (n = 1, Table 1) at these scan rates also confirms the absence of any contributions of the ECE type, or else from any product–substrate autocatalytic reactions [27,36,53]. However, at *v* < 0.05 V s^−1^, the apparent number of electrons for several silatranes tends to decrease (n → 0.5), which is indicative of a slow auto-protonation (*k*_p_) of the neutral **1** by its CR, similar to what was observed for trialkylamines [24,54,55,56] and for several parent germatranes [16,17]. Since silatranes are less basic (the issue of basicity of silatranes is more complex, because of the contributions from N and O centers [57]) than the parent Et_3_N and (HOCH_2_CH_2_)_3_N [1,58,59], their protonation is slower, so this current depletion appears at slower scan rates. This reaction refers to a CE scheme, and has no impact on *E*_p_. Since the protonation of **1** is slower than deprotonation of **1**^+•^ (*k*_p_ < *k*) within the time scale of CV, pure monomolecular deprotonation of **1**^+•^ remains the sole follow-up reaction of these transient species accessible via the *E*_p_ measurements.

The IR-corrected Δ*E*_p_/Δ*log*(*v*) plots (Figure 4) all show a slope close to 30 mV per decade of the *v* (Table 1) characteristic of monomolecular follow-up reactions of CRs [27], corroborating the notion that the deprotonation of CRs is the potential-determining step. As a relatively slow ET competes with the deprotonation at *v* > 1–5 V s^−1^, assessing the kinetic contribution in the EC scheme through the characteristic test *E*_p_ − *log*(*v*) can only be used within the 0.02 < *v* < 1–2 V s^−1^ scan rate range. DCV also confirmed a mixed diffusion–ET control at the scan rates *v* = 1…10 V s^−1^, such that the total chemical reversibility of oxidation of **1** (with *i*′_c_/*i*′_a_ = 1) could not be attained by increasing *v* (Figure 2B).

**Figure 9:**
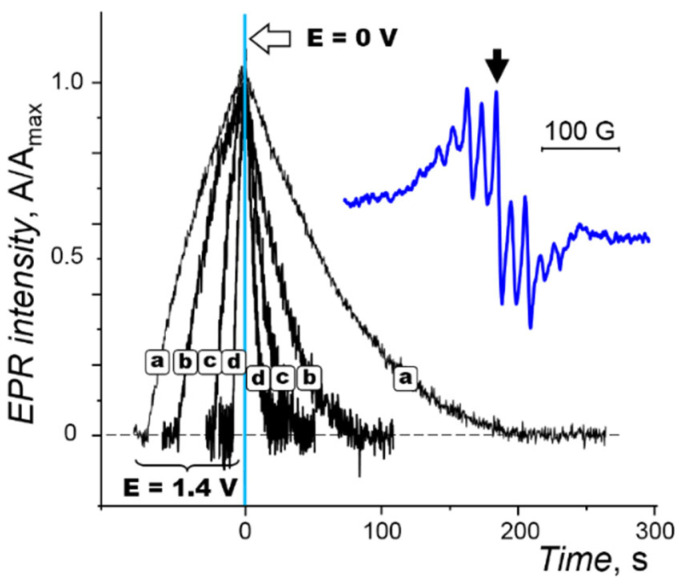
EPR spectrum of **1c**^+•^ (**1c** = 0.1 mmol L^−1^) generated at *E* = 1.4 V and its normalized decay at different temperatures monitored at the static field of the central line (3373 G, marked with an arrow). T = (**a**) 213 K, (**b**) 223 K, (**c**) 230 K, and (**d**) 240 K.

**Figure 10:**
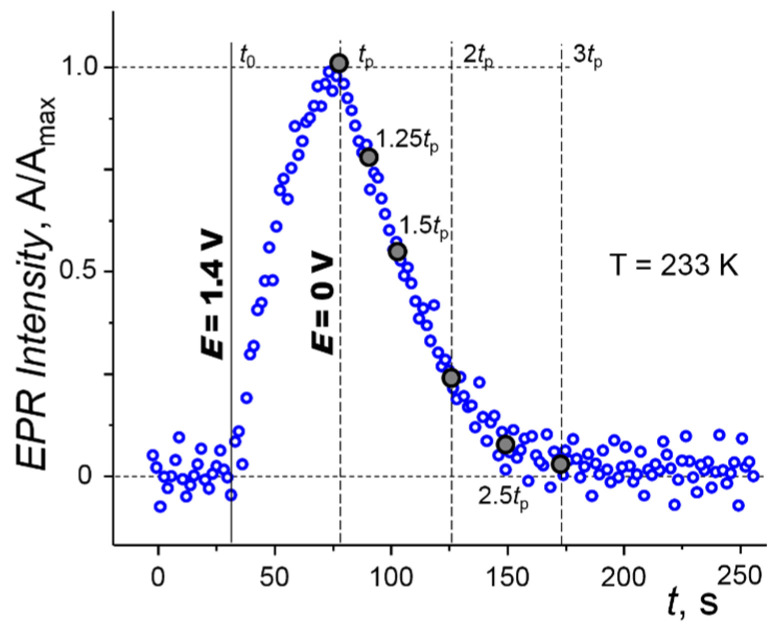
Generation and time decay of the normalized EPR signal of **1b**^+•^ and its sampling for *A*/*A*_max_ − *kjt*_p_^3/2^/FD^1/2^ analysis [60]. Pt micro spiral electrode, CH_3_CN/0.025 M Bu_4_NPF_6_, static field 3374 G, *f* = 9.46 GHz, modulation 1 G, Microwave power 6.2 mW, T = 233 K.

The intensity of the EPR signal of **1**^+•^ is directly related to the concentration of the latter, so that its decay can be used to obtain quantitative kinetic information on the follow-up chemical reactions of **1**^+•^ (Figure 9). First-order *log*(*A*/*A*_max_) = *f*(*t*) treatment and kinetic fitting of the decay curve according to [60] (Figure 10; see Section 3.) showed the potential-determining reaction of **1b**^+•^, **1c**^+•^, **1g**^+•^ and **1h**^+•^ to be of the first kinetic order. For instance, the deprotonation of the electrogenerated **1c**^+•^ was shown to occur with *k* = 0.28 s^−1^ at T = 298 K, which is in a good agreement with the CV data (Table 1). The variable temperature EPR of **1c**^+•^ (Figure 9) provided its activation enthalpy Δ*H*^≠^ = 3.94 kcal mol^−1^. Close values of the activation parameters and the deprotonation rate constants *k* were found for the CRs of **1g** (see also [11]), **1h** and **1b**. The average rates of deprotonation, determined on the basis of the results of CV, CA, and EPR spectroscopy, are collected in Table 1.

#### 2.3.2. Bimolecular Decay of **1**^+•^

Since bimolecular reactions involve molecular movements to bring the reagents together (Marcus work term w_R_ [61]), their kinetics is dependent on the diffusion coefficients D of the species involved. At the invariant atrane cage, the D of **1** is reciprocal to the bulkiness of the substituent R at the Si atom, and is higher for **1** with less encumbering substituents. It is probably for this reason that no indications of second-order reactivity could be observed for **1e**, **1g**, **1h** or **1i**. For **1b** with smaller R (R = Me), the poor reproducibility of the *E*_p_ − *log*(C) concentration tests, especially at C ≥ 2–3 mmol L^−1^, did not allow more reliable conclusions on this issue to be drawn.

In general, mechanistic criteria of CV provide much less support for the bimolecular reactivity of **1**^+•^. In fact, CR/CR dimerization (for all **1**^+•^, Δ*E*_p_/Δ*log*(*v*) ≥ 30 mV), as well as CR/substrate (Δ*E*_p_/Δ*log*(C) = 0) bimolecular reactions (for fast CR/substrate deprotonation, cf., e.g., [56,62,63]) can be excluded based on the criterion *E*_p_ = *f*(*v*), even though the latter process is of the first kinetic order on the CR and the condition Δ*E*_p_/Δ*log*(*v*) = 30 mV is respected. However, in the case of slow direct deprotonation (Equation (3)), **1**^+•^ (AH^+•^) might manifest their radical properties so that their disproportionation (Equation (5)) [36,38,53], followed by a sufficiently exergonic irreversible deprotonation (Equation (6)) of the resulting dication **1**^2+^ (AH^2+^) (i.e., ΔG_6_ < ΔG_5_), might become altogether favorable (Equation (7)).

(2) $$1\;\overset{-e}{\underset{Ep^{1}}{⇄}}\;1^{+•}\;({\mathrm{AH}}^{+•})$$

(3) $${\mathrm{AH}}^{+•}\;\underset{k}{\to }{\;A}^{•}+{\;H}^{+}$$

(4) $${1+H}^{+}\underset{k_{p}}{\to }{\mathrm{AH}}_{2}^{+}(\mathrm{redox}\;\mathrm{inactive})$$

(5) $${2\mathrm{AH}}^{+•}\;\underset{k_{disp}}{⇄}{\;1+\mathrm{AH}}^{2+}$$

(6) $${\mathrm{AH}}^{2+}\;\underset{k’}{\to }{\;A}^{+}{+H}^{+}$$

(7) $${2\mathrm{AH}}^{+•}\;\underset{}{\to }{\;1+A}^{+}{+H}^{+}$$

Since AH^2+^ can arise either from disproportionation (Equation (5)) (EC_disp_C_deprot_ process) or from the direct oxidation of AH^+•^ at *E*_p_^2^ (for several **1**, a second oxidation peak was observed at *E*_p_^2^ = *E*_p_^1^ + 300… 400 mV), the feasibility of this process can be evaluated by considering Equations (5)–(7) within the thermochemical Scheme (8).

(8) $$\begin{array}{lll} {\;\;\;\;\mathrm{AH}}^{+•} & \overset{-e}{\underset{E_{\left({\mathrm{AH}}^{2+}/{\mathrm{AH}}^{+}\right)}^{0}}{\to }} & {\;\;\;\;\mathrm{AH}}^{2+}\\ -H^{+}↓△G_{\left(3\right)} & & △G_{\left(6\right)}↓-H^{+}\\ {\;\;\;\;\;A}^{•} & \overset{-e}{\underset{E_{\left(A^{+}/A^{•}\right)}^{0}}{\to }} & {\;\;\;\;\;A}^{+} \end{array}$$

Assuming, in the first approach, *E*_p_^2^ ≅ *E*_0_(AH^2+^/AH^+•^), the ΔG_(6)_ of the deprotonation of AH^2+^ can be defined as ΔG_(6)_ = ΔG_1_ + F(*E*_p_^2^ – *E*_0_(A^+^/A•)). Free radicals are usually easier to oxidize than positively charged species, so one might expect that *E*_0_(A^+^/A•) < *E*_p_^2^, and therefore that ΔG_(6)_ > ΔG_(3)_. Direct evaluation of the thermochemistry of (5)–(7) for **1c**^+•^ (at the B3PW91/6-311++G(d,p) level of theory, C-PCM in CH_3_CN) corroborates this, providing −25.75 kcal mol^−1^ for the Gibbs energy of the deprotonation of **1c**^2+^, while for the disproportionation alone (Equation (5)), it only amounts to ΔG_(5)_ = −0.96 kcal mol^−1^. For model **1a**, this trend is even more pronounced (Table 2).

By comparing *k*_s_ and *k* (Table 1), it can be suggested that more stable CRs (formed with lower reorganization energy) might tend to disproportionation/dimerization reactions, while less stable CRs follow direct deprotonation.

### 2.4. DFT Calculations

Bond stretch isomerism in the CRs of silatranes [14] and the above experimental data imply that, from a practical point of view, it is virtually impossible to obtain primary **1**^+•^ in the geometry of the neutral **1** (and hence to observe Nernstian reversible voltammograms), because ET in such systems intrinsically involves a skeletal rearrangement caused by the destruction of the bonding 3c-4e N→Si internal system upon the removal of one electron.

Resulting from the withdrawal of one electron, the 3c-3e system no longer has the bonding character of the initial 3c-4e hyperbond, so the **1**^+•^_endo_ flops to its *exo* configuration **1**^+•^_exo_ (corresponding to *short* and *long* bond stretch isomers of **1**^+•^ [14]). However, at low scan rates, when ET does not limit the overall rate of the process, the first step of oxidation appears to be reversible for most of **1** and quasi-reversible for the others by means of a common follow-up chemical step—first-order deprotonation (see above).

A quantitative parameter characterizing the equilibrium of deprotonation between **1**^+•^ and **1**^•^ (and generally, for any CRs: AH^+•^ Δ A^•^ + H^+^) is the value of p*K*_a_ (Table 3). The greater the value of p*K*_a_, the higher the stability of the corresponding CR.

Corroborating the nature of the decay of the CRs—the loss of H^+^—the experimental kinetics of disappearance of **1**^+•^, determined by the electrochemical methods, closely parallels (Brønsted plot, n = 4, R = 0.997, *p* = 0.00284) their thermodynamic acidity, obtained from B3PW91/6-311++G(d,p) (acetonitrile, C-PCM) quantum chemical calculations (Equation (9)):

*log*(k) = 0.258 × p*K*_a_ − 1.436 (9)

Going through the p*K*_a_ of silatranes **1** (Table 3), an interesting trend can be observed, whereby whatever the substituent R, the proton in **1**^+•^ is preferentially eliminated from β-carbon. This finding is utterly surprising, because according to the literature [23,24,64], the deprotonation of CRs of alkyl amines occurs at α-carbon.

It should be noted that the oxidation of vinyl silatrane has been reported in the presence of nucleophilic CN^-^ anion, and the reaction site undergoing oxidative deprotonation–cyanation was then suggested to be the α-carbon [15]. However, the conditions reported seem not to be adapted for a selective anodic cyanation. This process works smoothly with tertiary amines at remarkably lower potentials [64], while *E*_p_(**1c**) is anodically shifted by +0.8 V compared to *E*_p_(Et_3_N) because of the N→Si donation [6], such that the oxidation of CN^−^ to give CN^•^ interferes with the oxidation of **1c**, complicating the whole mechanism. Our attempt to reproduce cyanation under the conditions reported in [15] yielded a mixture of at least four cyanated products that were hard to discern based only on NMR data.

**Figure 11:**
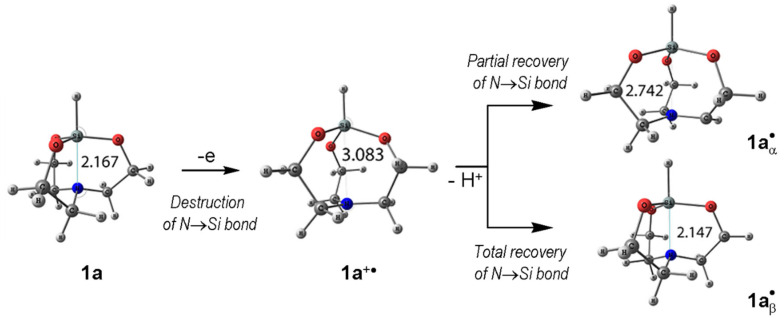
Variation in the length (Å) of dative contact N → Si when going from neutral **1a** to its cation radical **1a**^+•^ and further to the deprotonated forms **1a**^•^_α_ and **1a**^•^_β_. From DFT B3PW91/6-311++G(d,p) in acetonitrile (C-PCM).

What might be the reason for these particularities of the deprotonation of **1**^+•^? In order to answer this question, the variation in the length of the coordination contact N→Si (*l*_Si-N_) during the process **1a** → **1a**^+•^ → **1a**^•^_α_ (**1a**^•^_β_) in CH_3_CN should be considered (Figure 11).

A dramatic increase (by ~0.94 Å) in the distance N···Si occurs at the stage **1a** → **1a**^+•^ corresponding to the destruction of N→Si bond. At the deprotonation step, a ca. 0.34 Å shortening accompanies the transition **1a**^+•^  **→ 1a**^•^_α_, while a spectacular shortening (by 0.95 Å) occurs when going from **1a**^+•^ to **1a**^•^_β_ (Figure 11, Table 3). This unequivocally attests to a partial restoration of the N→Si bonding in radicals **1**^•^_α_ and its total restoration in **1**^•^_β_. As a result, **1**^•^_β_ is thermodynamically favored over **1**^•^_α_ (see Δ*G*_β-α_ in Table 3).

Therefore, the low stability of the CRs of silatranes towards deprotonation—as compared to the CRs of amines—arises from the stabilization of their decomposition products (**1**^•^_α_ and, in particular, **1**^•^_β_) due to the coordination interaction N→Si and the restitution of the 3c-4e bond in these secondary species.

## 3. Experimental Section

The studied silatranes were prepared from the corresponding R-Si(OEt)_3_ precursors and N(CH_2_CH_2_OH)_3_ according to the method described in [11]. CH_3_CN was distilled from CaH_2_ and stored over 4 Å molecular sieves, Bu4NPF6 (Aldrich) was vacuum-dried for 10 h at 80–90 °C prior to use.

Cyclic (CV), square-wave cyclic (SWCV) voltammetry, two-step potentiostatic chronoamperometry (CA) and electrochemical impedance spectroscopy (EIS) were carried out using a PAR 2273 scanning potentiostat operating under PowerSuite (PAR, release 2.58) software. A 25 mL cell was used with a Pt (0.5 mm) disk working electrode in a three-electrode configuration. Ag wire, electrolytically covered with AgCl, was used as a reference electrode; all measured potentials were corrected with respect to the *E*_0_(Fc^+^/Fc) of ferrocene (0.431 V vs. Ag/AgCl under the conditions given or 0.31 V vs. SCE [21]). The IR-compensation of ohmic drops in a CH3CN/0.1M Bu4NPF6 solution was applied in all experiments; all measurements were carried out under argon.

The Butler–Volmer equation for small overpotentials (ΔE = 10 mV) was used to determine the apparent heterogeneous ET rate constant *k*_s_ using charge transfer resistance R_CT_ from EIS, under the assumption of similar diffusion coefficients for the neutral silatrane and its CR (D_AH_^+•^ = D_AH_ = D). Diffusion-free zero-Warburg resistance, R_W0_, defined as R_W0_ = R_CT_ − R_CT_^2^CDL(*k*_f_/D_ox_^1/2^ + *k*_b_/D_red_^1/2^)^2^ [41], was obtained from the intersection of the linear Warburg impedance slope (at *f* → ∞) with the Z_R_′ axis. Assuming the equilibrium potential *k*_f_ = *k*_b_ = *k*_s_ defines (with the experimental R_W0_, R_CT_ and C_DL_, and D obtained using the Einstein–Stokes equation) the ET rate as follows [41]:

*k*_s_ = (*k*_f_ + *k*_b_)/2 = 0.5[(R_CT_ − R_W0_)D/R_CT_^2^C_DL_]^1/2^ (10)

When the reduction peaks of CRs appeared at *v* ≅ 0.1 V s^−1^ (kinetic parameter λ = *k*RT/nF*v* falling in the interval 4 × 10^−3^ < λ < 20 [27]), the *k* of deprotonation was directly derived using Equation (11) [38]:

*E*_p_ = *E*_0_ + 0.78RT/nF − (RT/2nF)*ln*(λ) (11)

When the *i*_p_^a^/*i*_p_^c^ ratio in CV fell in the range between 0.25 (**1d**) and 0.59 (**1h**) at 1 V s^−1^, the rate constant *k* was determined using the working curve *i*_p_^a^/*i*_p_^c^ − *log*(*k*τ) (τ is the time elapsed during the scan between *E*_1/2_ and the vertex potential) [36]. For ill-shaped CV peaks, when measuring the *E*_p_ is difficult, the derived CV (DCV [27]) and two-step potentiostatic chronoamperometry (CA [36]) were used to assess *k* using inflection potential *E*_i_ for *E*_p/2_ and peak current *i*′ [65].

EPR spectra were registered on a Bruker EMX 300 (X-band) spectrometer with a Gunn diode coupled with a standard rectangular cavity at 9.46 GHz, MW power 2–8 mW and modulation frequency 100 kHz. Winsim-2002 [66] was used for EPR spectra simulation.

Quantum chemical calculations (geometry optimization and harmonic frequency analysis) were carried out with Gaussian-09 [67] using DFT (B3PW91) methods with the basis set 6-311++G(d,p), while applying the C-PCM solvation model [68]. The literature [69] attests that this method perfectly reproduces the known experimental gas-phase (electron diffraction) geometries of the “common” silatranes X-Si(OCH_2_CH_2_)_3_N (X = H, Me, F). The value of the mean arithmetic error (MAE = 0.02) when using the B3PW91 method for describing the Si···N contact length in these compounds suggests that this method is almost as good as high-precision CCSD due to the lucky compensation of errors.

Deprotonation can be quantitatively described via the logarithm of the dissociation constant *K*_a_ (Equation (12) [70,71]):

p*K*_a_ = −*log K*_a_ = ΔG*_solv_(AH^+•^)/(2.3 *RT*) (12)

where ΔG*_solv_(AH^+•^) is the free energy of deprotonation of AH^+•^ in solution:

ΔG*_solv_ = *G*_solv_^A•^ + *G*_solv_^H+^ − *G*_solv_^AH+•^ (13)

with *G*_solv_^A+•^, *G*_solv_^H+^ and *G*_solv_^AH+•^ being the free energies of the corresponding species in solution.

The term *G*_solv_^H+^ is defined as:

*G*_solv_^H+^ = *G*_gas_^H+^ + Δ*E*_solv_^H+^ + Δ*G*_gas → solv_. (14)

The free energy of protons in the gas phase (*G*_gas_^H+^) is −6.28 kcal mol^−1^ [70]. The solvatation energy in acetonitrile (Δ*E*_solv_^H+^) was taken to be −255.1 kcal mol^−1^ [72]. An additional energy contribution, Δ*G*_gas → solv_ = −1.9 kcal mol^−1^, accounts for the transfer of H^+^ from the gas phase (1 atm) to 1 mol of solvent in its standard state [70].

## 4. Conclusions

The formation of the short-lived cation radicals during the oxidative ET from silatranes **1** was unequivocally established. The formation of these CRs involves a substantial skeletal reorganization, limiting the ET rate and destroying N→Si dative coordination and consequently the whole internal 3c-4e bonding system. At sufficiently long time scales (low scan rates *v* in CV), ET is close to Nernstian; at shorter time scales (higher *v*), ET becomes the limiting step, which corresponds to the experimental ΔG^≠^ and reorganization energies *λ*. The data of various electrochemical methods and of EPR spectroscopy unambiguously demonstrate deprotonation to be the main path of decay of the CRs of silatranes. The DFT B3PW91/6-311++G(d,p) analysis suggests the preferred reaction pathway of silatrane CRs to be the elimination of H^+^ from the β-CH_2_ group of the atrane cage. This reaction path, in contrast to α-deprotonation, allows the atrane nitrogen to recover its lone electron pair and to restore the 3c-4e hyperbond and N→Si coordination, thus providing substantial stabilization to the resulting radical.

From the experimental and theoretical findings of this work, the stability of the CRs of silatranes against deprotonation can be expected to be enhanced upon:

(1) Weakening of the N→Si interaction in the starting silatranes (i.e., reducing the electronegativity of the substituent R and the solvent polarity); and
(2) Replacing the protons at C^α^ and C^β^ atoms with alkyl groups or with F atoms, or else merging the C^α^–C^β^ bond into side cyclic structures, primarily aromatic (phenylene substitution). The latter means passing to tribenzsilatranes (see [43,73,74]), in which the deprotonation is expected to be less important due to the unfavorable orbital orientation of the p_z_(N) and π systems of the side aromatic rings, reducing positive charge delocalization in the latter in the CRs. Further study in these directions is underway and will be published elsewhere.

## Figures and Tables

**Table 1:** Parameters of electrooxidation of silatranes **1** in CH_3_CN/0.1 Bu_4_NPF_6_ at a Pt electrode (*v* = 0.1 V s^−1^) ^a^.

Cpd	R	n	*E*_p_	*E*_0_	*E*_p_–*E*_p/2_, ^b^	Δ*E*_p_/Δ*log*(*v*) ^b,c^	*k*_s_, cm s^−1^	*k*, s^−1 d^
**1b**	Me	0.9	1.43	1.42	68	30	0.272	0.16 (0.33)
**1c**	CH = CH_2_	1.0	1.52	1.49	102	35	0.078	0.30 (0.28)
**1d**	(CH_2_)_2_CN	0.9	1.45	1.38	81	32	0.046	0.66
**1e**	CH_2_Ph	1.0	1.30	1.27	61	40	0.068	1.47
**1f**	CH_2_(C_10_H_7_)	1.0	1.10	-	-	-	-	-
**1g**	Ph	1.0	1.53	1.50	85	34	0.156	0.43 (0.33) ^e^
**1h**	C_6_H_4_CH_3_	1.0	1.56	1.53	65	29	0.180	0.40 (0.54)
**1i**	C_6_H_4_Cl	1.0	1.60	1.55	75	35	0.053	2.24
**1j**	Cl	0.9	2.05	2.01	87	-	0.003	27

^a^ *E*, V vs. SCE. ^b^ mV. ^c^ Δ*E*_p_/Δ*log*(*C*) ≅ 0 for silatranes **1**. ^d^ From CV and CA; in parentheses the values from EPR spectroscopy. ^e^ From Romanovs, V., et al. 2017 [11].

**Table 2:** Free Gibbs energy ΔG (kcal mol^−1^) of disproportionation of **1a**^+•^ and **1c**^+•^ and of the following deprotonation of the dications **1c** ^2+^ and **1c** ^2+^. From B3PW91/6-311++G(d,p) in CH_3_CN (C-PCM).

Silatrane	ΔG_(5)_	ΔG_(6)_	ΔG_(7)_
**1a**	−3.25	−30.75	−34.00
**1c**	−0.96	−25.75	−26.71

**Table 3:** Interatomic distances N→Si (*l*_Si-N_, Å) in silatranes **1**, in their CRs **1**^+•^ and in radicals **1**^•^_α_ and **1**^•^_β_; calculated equilibrium constants p*K*_a_ (deprotonation **1**^+•^ ⇄ **1**^•^ + H^+^); and the relative stability of the two deprotonated forms, **1**^•^_α_ and **1**^•^_β_ (Δ*G*_β-α_ = *G*(**1**^•^_β_) − *G*(**1**^•^_α_), kcal mol^−1^). From B3PW91/6-311++G(d,p) in acetonitrile solution (C-PCM).

Cpd	R	*l*_Si-N_(1)	*l*_Si-N_(1^+•^)	*l*_Si-N_(1^•^_α_)	*l*_Si-N_(1^•^_β_)	p*K*_a_(C^α^)	p*K*_a_(C^β^)	Δ*G*_β-α_
**1a**	H	2.167	3.083	2.742	2.147	7.40	4.47	−3.99
**1b**	Me	2.269	3.118	2.924	2.233	9.46	8.57	−1.21
**1c**	Vinyl	2.232	3.109	2.885	2.206	8.87	7.36	−2.07
**1g**	Ph	2.239	3.106	2.897	2.210	8.94	7.81	−1.54
**1j**	Cl	2.099	3.050	2.123	2.087	2.91	0.03	−3.94

## Data Availability

Not applicable.

## References

[1] Frye C.L., Vogel G.E., Hall J.A. (1961). Triptych-Siloxazolidines: Pentacoordinate bridgehead silanes resulting from transanular interaction of nitrogen and silicon. J. Am. Chem. Soc..

[2] Pestunovich V., Kirpichenko S., Voronkov M., Rappoport Z., Apeloig Y. (1998). Silatranes and their tricyclic analogs. Chemistry of Organic Silicon Compounds.

[3] Voronkov M.G., D’yakov V.M., Kirpichenko S.V. (1982). Silatranes. J. Organomet. Chem..

[4] Pombeiro A.J.L., McCleverty J.A. (1993). Molecular Electrochemistry of Inorganic, Bioinorganic and Organometallic Compounds.

[5] Broka K., Glezer V., Stradiņš J., Zelčāns G. (1991). Anodic oxidation of silatrane and its Si-substituted derivatives in acetonitrile at a graphite electrode. J. Obsch. Khim..

[6] Broka K., Stradiņš J., Glezer V., Zelčāns G., Lukevics E. (1993). Electrochemical oxidation of silatranes. J. Electroanal. Chem..

[7] Sidorkin V.F., Pestunovich V.A., Voronkov M.G. (1977). The structure of silatranes from the theory of hypervalent bonding. Dokl. Akad. Nauk SSSR.

[8] Peureux C., Jouikov V. (2014). Covalent Grafting of Silatranes to Carbon Interfaces. Chem.-A Eur. J..

[9] Zhao L., Pan S., Holzmann N., Schwerdtfeger P., Frenkin G. (2019). Chemical Bonding and Bonding Models of Main-Group Compounds. Chem. Rev..

[10] Carey F.A., Sundberg R.G. (2007). Advanced Organic Chemistry.

[11] Romanovs V., Sidorkin V.F., Belogolova E.F., Jouikov V. (2017). Cation Radical of Phenylsilatrane. Dalton Trans..

[12] Wang Y. (2012). Synthèse, Réactivité Electrochimique et Structure Electronique de 1-R-métallatranes (M = Si, Sn). Ph.D. Thesis.

[13] Meshgi M.A., Baumgartner J., Jouikov V.V., Marschner C. (2017). Electron transfer and modification of oligosilanylsilatranes and related derivatives. Organometallics.

[14] Sidorkin V., Belogolova E.F., Wang Y., Jouikov V., Doronina E.P. (2017). Electrochemical oxidation and cation radicals of structurally non-rigid hypervalent silatranes. Theoretical and experimental studies. Chem. Eur. J..

[15] Soualmi S., Ignatovich L., Lukevics E., Ourari A., Jouikov V. (2008). Electrochemical oxidation of benzyl germatranes. J. Organomet. Chem..

[16] Soualmi S., Ignatovich L., Jouikov V. (2010). Cation radicals of organogermatranes. Appl. Organomet. Chem..

[17] Ignatovich L., Jouikov V. (2014). Organogermatranes and their cation radicals by EPR-spectroelectrochemistry and ab initio calculations. J. Organomet. Chem..

[18] Zoller T., Dietz C., Iovkova-Berends L., Karsten O., Bradtmoeller G., Wiegand A.-K., Wang Y., Jouikov V., Jurkschat K. (2012). Novel Stannatranes of the Type N(CH_2_CMe_2_O)_3_SnX (X = OR, SR, OC(O)R, SP(S)Ph_2_, Halogen). Synthesis, Molecular Structures and Electrochemical Properties. Inorg. Chem..

[19] Meshgi M.A., Pöcheim A., Baumgartner J., Jouikov V.V., Marschner C. (2021). Oligosilanylated Silocanes. Molecules.

[20] Lutter M., Iovkova-Berends L., Dietz C., Jouikov V., Jurkschat K. (2012). N-Aryl-substituted 5-aza-2,8-dioxasilabicyclo[3.3.01.5]octanes: Syntheses, molecular structures, DFT calculations and cyclovoltammetric studies. Main Group Met. Chem..

[21] Mann C.K., Barnes K.K. (1970). Electrochemical Reactions in Nonaqueous Systems.

[22] Chow Y.L., Danen W.C., Nelsen S.F., Rosenblatt D.H. (1978). Nonaromatic Aminium Radicals. Chem. Rev..

[23] Nelsen S.F., Kessel C.R., Brien D.J. (1980). Bredt’s rule kinetically stabilized nitrogen-centered radical cations and radicals in the 9-azabicyclo[3.3.1]nonyl system. J. Am. Chem. Soc..

[24] Nelsen S.F., Ippoliti J.T. (1986). The deprotonation of trialkylamine cation radicals by amines. J. Am. Chem. Soc..

[25] Dinnocenzo J.P., Banach T.E. (1989). Deprotonation of tertiary amine cation radicals. A direct experimental approach. J. Am. Chem. Soc..

[26] Nelsen S.F., Cunkle G.T. (1985). Long-lived trialkylamine radical cations containing alpha-carbon-hydrogen bonds in acyclic alkyl groups. J. Org. Chem..

[27] Onomura O., Hammerich O., Speiser B. (2016). Organic Electrochemistry, Revised and Expanded.

[28] Moeller K.D., Schaefer H.J. (2004). Organic Electrochemistry. Encyclopedia of Electrochemistry.

[29] Steckhan E., Lund H., Hammerich O. (2001). Organic Electrochemistry.

[30] Hammerich O., Utley J.H.P., Eberson L., Speiser B., Hammerich O. (2001). Organic Electrochemistry.

[31] Lewis F.D., Ho T.-I., Simpson J.T. (1981). Photochemical addition of tertiary amines to stilbene. Stereoelectronic control of tertiary amine oxidation. J. Org. Chem..

[32] Lewis F.D., Ho T.-I. (1977). Trans-Stilbene-amine exciplexes. Photochemical addition of secondary and tertiary amines to stilbene. J. Am. Chem. Soc..

[33] Kanoufi F., Zu Y., Bard A.J. (2001). Electrogenerated chemiluminescence. IX. Electrochemistry and emission from systems containing tris(2,2′-bipyridine)ruthenium(II) dichloride. J. Phys. Chem..

[34] Adenier A., Chehini M.M., Gallardo I., Pinson J., Vila N. (2004). Electrochemical oxidation of aliphatic amines and their attachment to carbon and metal surfaces. Langmuir.

[35] Yang M., Albrecht-Schmitt T., Cammarata V., Livant P., Makhanu D.S., Sykora R., Zhu W. (2009). Trialkylamines More Planar at Nitrogen Than Triisopropylamine in the Solid State. J. Org. Chem..

[36] Bard A.J., Faulkner L.R. (2001). Electrochemical Methods, Fundamentals and Applications.

[37] Navarro-Suarez A.M., Johansson P. (2019). A silatrane: Molecule-based crystal composite solid-state electrolyte for all-solid-state lithium batteries. Batter. Supercaps.

[38] Savéant J.-M., Costentin C. (2019). Elements of Molecular and Biomolecular Electrochemistry: An Electrochemical Approach to Electron Transfer Chemistry.

[39] Bond A.M., Henderson T.L.E., Mann D.R., Mann T.F., Thormann W., Zoski C.G. (1988). Fast Electron Transfer Rate for the Oxidation of Ferrocene in Acetonitrile or Dichloromethane at Platinum Disk Ultramicroelectrodes. Anal. Chem..

[40] Pavlishchuk V.V., Addison A.W. (2000). Conversion constants for redox potentials measured versus different reference electrodes in acetonitrile solutions at 25 °C. Inorganica Chim. Acta.

[41] Lvovich V.F. (2012). Impedance Spectroscopy.

[42] Doronina E.P., Sidorkin V.F., Lazareva N.F. (2010). (PO→Si) Chelates of Silylmethyl Derivatives of Phosphoric Acids R_2_P(O)ZCH_2_SiMe_3−*n*_Hal*_n_* (*n* = 1−3; Z = O, NMe, CH_2_, S). Organometallics.

[43] Jouikov V. (2010). Electrochemical Oxidation and Cation Radicals of All-Five and All-Six 1-Substituted Metallatranes (M = Si, Ge). Spectroelectrochemical Study. ECS Trans..

[44] Fessenden R.W., Neta P.J. (1972). Electron spin resonance spectra of di- and trimethylaminium radicals. J. Phys. Chem..

[45] De Meijere A., Chaplinski V., Gerson F., Merstetter P., Haselbach E. (1999). Radical Cations of Trialkylamines:  ESR Spectra and Structures. J. Org. Chem..

[46] Eastland G.W., Rao D.N., Symons M.C.R. (1984). Amine Radical Cations: An Electron Spin Resonance Study of Cations generated by Radiolysis. J. Chem. Soc. Perkin Trans..

[47] Boese R., Blaer D., Antipin M.Y., Chaplinski V., de Meijere A. (1998). Non-planar structures of Et_3_N and Pr^i^_3_N: A contradiction between the X-ray, and NMR and electron diffraction data for Pr^i^_3_N *Chem*. Commun..

[48] Gerson F., Huber W. (2003). Electron Spin Resonance Spectroscopy of Organic Radicals.

[49] Sterkhova I.V., Lazarev I.M., Smirnov V.I., Lazarev N.F. (2015). 1-(Methylaminomethyl) silatrane: Synthesis, characterization and reactivity. J. Organomet. Chem..

[50] Pestunovich V.A., Sidorkin V.F., Dogaev O.B., Voronkov M.G. (1980). Order of the X-SiN hypervalent bond in silatrane molecules. Dokl. RAS.

[51] Romanovs V., Spura J., Jouikov V. (2018). Synthesis and electrochemical study of 1,1′-thienyl-substituted fused bis-germatranes with a core 5c-6e hyperbond. Synthesis.

[52] Smallwood I.M. (1996). Handbook of Organic Solvent Properties.

[53] Pedersen S.U., Daasbjerg K., Balzani V. (2001). Electron Transfer in Chemistry.

[54] Vyushkova M.M., Bagryansky V.A., Molin Y.N. (2010). Application of Optically Detected EPR to Study the Stability of Sterically Hindered Amine Radical Cations to Proton Transfer Reaction. Appl. Magn. Reson..

[55] Belevskii V.N., Belopushkin S.I., Nuzhdin K.B. (2007). ESR study of the mechanism of radical formation during liquid-and solid-phase radiolyses of ethylamines. High Energy Chem..

[56] Lefkowitz S.M., Trifunac A.D. (1984). Time-resolved fluorescence-detected magnetic resonance and fluorescence studies of trialkylamines irradiated by pulse radiolysis in alkane solvents. J. Phys. Chem..

[57] Garant R.J., Daniels L.M., Das S.K., Janakiraman M.N., Jacobson R.A., Verkade J.G. (1991). Electronic tuning of asymmetric catalysts. J. Am. Chem. Soc..

[58] Yashikawa A., Gordon M.S., Sidorkin V.F., Pestunovich V.A. (2001). Proton affinities of the Silatranes and Their Analogues. Organometallics.

[59] Voronkov M.G., Belyaeva V.V., Abzaeva K.A. (2012). Basicity of Silatranes (Review). Chem. Heterocycl. Compd..

[60] Goldberg I.B., Boyd D., Hirasawa R., Bard A.J. (1974). Simultaneous electrochemical-electron spin resonance measurements. III. Determination of rate constants for second-order radical anion dimerization. J. Phys. Chem..

[61] Marcus R.A. (1993). Electron transfer reactions in chemistry: Theory and experiment. Angew. Chem. Int. Ed. Engl..

[62] Vesala A., Lonnberg H. (1980). Salting-in Effects of Alkyl-Substituted Pyridinium Chlorides on Aromatic Compounds. Acta Chem. Scand. A.

[63] Oyamaa M., Higuchi T. (2002). Electron-Transfer Stopped-Flow Method: Its Validity for Spectrochemical Analysis of Electrogenerated Cation Radicals. J. Electrochem. Soc..

[64] Louafi F., Moreau J., Shahane S., Golhen S., Roisnel T., Sinbandhit S., Hurvois J.-P. (2011). Electrochemical Synthesis and Chemistry of Chiral 1-Cyanotetrahydroisoquinolines. An Approach to the Asymmetric Syntheses of the Alkaloid (−)-Crispine A and Its Natural (+)-Antipode. J. Org. Chem..

[65] Espinoza E.M., Clark J.A., Soliman J., Derr J.B., Morales M., Vullev V.I. (2019). Practical Aspects of Cyclic Voltammetry: How to Estimate Reduction Potentials When Irreversibility Prevails. J. Electrochem. Soc..

[66] O’Brien D.A., Duling D.R., Fann Y.C. (2002). WinSim-2002, PEST Version 0.98.

[67] Frisch M.J., Trucks G.W., Schlegel H.B., Scuseria G.E., Robb M.A., Cheeseman J.R., Scalmani G., Barone V., Mennucci B., Petersson G.A. (2010). Gaussian 09, Revision C.01.

[68] Tomasi J., Mennucci B., Cammi R. (2005). Quantum Mechanical Continuum Solvation Models. Chem. Rev..

[69] Belogolova E.F., Shlykov S.A., Eroshin A.V., Doronina E.P., Sidorkin V.F. (2021). The hierarchy of ab initio and DFT methods for describing an intramolecular non-covalent Si···N contact in the silicon compounds using electron diffraction geometries. Phys. Chem. Chem. Phys..

[70] Houari Y., Jacquemin D., Laurent A.D. (2013). Methodological keys for accurate pK*_a_ simulations. Phys. Chem. Chem. Phys..

[71] Keith J.A., Carter E.A. (2012). Quantum Chemical Benchmarking, Validation, and Prediction of Acidity Constants for Substituted Pyridinium Ions and Pyridinyl Radicals. J. Chem. Theory Comput..

[72] Rossini E., Knapp E.-W. (2016). Proton Solvation in Protic and Aprotic Solvents. J. Comput. Chem..

[73] Frye C.L., Vincent G.A., Hauschildt G.L. (1966). Pentacoordinate Silicon Derivatives. 111. 2,2’,2”-Nitrilotriphenol, a New Chelating Agent. J. Am. Chem. Soc..

[74] Meshgi M.A., Zaitsev K.V., Vener M.V., Churakov A.V., Baumgartner J., Marschner C. (2018). Hypercoordinated Oligosilanes Based on Aminotrisphenols. ACS Omega.

